# P-1306. Establishing Respiratory Syncytial Virus (RSV) Surveillance Methodology within the Indian Health Service

**DOI:** 10.1093/ofid/ofae631.1487

**Published:** 2025-01-29

**Authors:** Keya Desai, Andria Apostolou, Sarah Houghton, Uzo Chukwuma

**Affiliations:** Indian Health Service, Shrewsbury, Massachusetts; Indian Health Service, Shrewsbury, Massachusetts; Indian Health Service, Shrewsbury, Massachusetts; Navy and Marine Corps Public Health Center, Rockville, Maryland

## Abstract

**Background:**

American Indian and Alaska Native (AI/AN) populations have high rates of Respiratory Syncytial Virus (RSV)-associated hospitalizations among both infants and older populations. Due to underrepresentation, most national surveillance reports do not adequately describe the burden of disease in the AI/AN population. Currently, there is no standardized approach for RSV surveillance within the IHS. This work explores establishing RSV surveillance using IHS-specific electronic medical records (EMR) data. Additionally, this work aims to implement a standardized surveillance methodology across IHS for RSV and adaptable for other infectious diseases. The objective of this abstract is to describe the process of RSV surveillance methodology within the IHS.

Venn diagram of ICD and LOINC datasets

**Figure:**
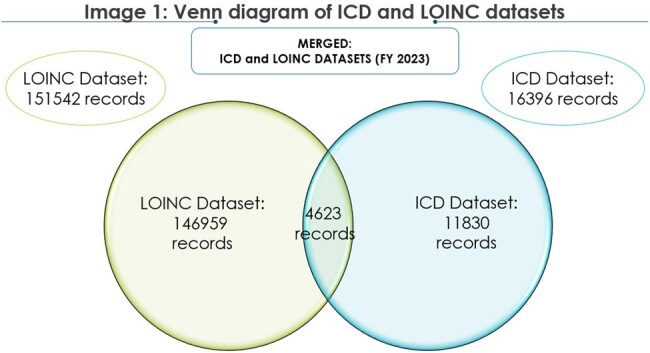

The above image is a representation of the Venn diagram of ICD and LOINC datasets. The two datasets are merged together to identify the overlapped records and unique ICD and LOINC records for RSV.

**Methods:**

We investigated the utility of using administrative data from Federal, Tribal, and Urban Indian health facilities for RSV surveillance among AI/AN population for Fiscal Year (FY) 2023. We evaluated RSV administrative codes, such as Logical Observation Identifiers Names and Codes (LOINC), International Classification of Diseases (ICD) 9 and 10, to develop an algorithm for RSV records. The records that contained either an ICD 9 or ICD 10 code for RSV came from the ICD dataset; records that contained RSV-specific LOINC code came from the LOINC dataset. Final set of records were flagged as positive and negative RSV records.

**Table 1.**
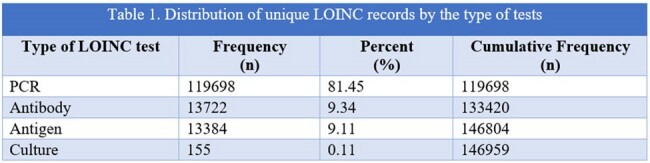
Distribution of unique LOINC records by the type of tests

The table shows the distribution of the unique LOINC records by the four categories of type of tests. The categories include PCR, Antigen, Antibody and Culture tests.

**Results:**

In FY 2023, 16396 records came from the ICD dataset and 151542 records came from the LOINC dataset. On merging ICD and LOINC datasets, 4623 records overlapped, 11830 records were unique to ICD dataset and 146959 records were unique to LOINC dataset. Of the records unique to LOINC dataset (146959), 81% (n=119698) represent PCR tests while antigen (n=13722) and antibody (n=13384) tests represent 9% respectively.

**Conclusion:**

This study highlights the importance of using both ICD and LOINC codes for identifying RSV cases in EMR. It aids in understanding trends and burden among AI/AN population. Currently, we are conducting site validations to evaluate the surveillance methods across IHS. Additionally, surveillance reports and epidemiological studies generated using this standard approach could help target interventions for RSV and other infectious diseases.

**Disclosures:**

**All Authors**: No reported disclosures

